# A longitudinal study on the professional identity formation of health promotion practitioners: evidence from undergraduate students in Switzerland

**DOI:** 10.3389/fmed.2025.1491467

**Published:** 2025-02-10

**Authors:** Verena Biehl, Andreas Bänziger, Frank Wieber

**Affiliations:** ^1^School of Health Sciences, Institute of Public Health, ZHAW Zurich University of Applied Sciences, Winterthur, Switzerland; ^2^Faculty of Health Sciences, School of Public Health, Bielefeld University, Bielefeld, Germany; ^3^Department of Psychology, University of Konstanz, Konstanz, Germany

**Keywords:** health promotion, public health, workforce, professional identity, capacity building, undergraduate students

## Abstract

**Introduction:**

Professional identity (PI) is crucial for workforce capacity building, as it leads to the adoption of the professional role and commitment. And yet, there is little literature on the PI of health promotion practitioners as part of the public health workforce. Education plays a significant role in PI formation. Therefore, the aim of this study is to investigate PI formation in undergraduate health promotion students. To conceptualize PI, we draw on social psychological theories and consider potential determinants across cognitive, social, motivational, and behavioral dimensions.

**Methods:**

To gain insights into the PI formation of health promotion students, an observational longitudinal study was conducted using an online survey at three times of measurement. Undergraduate physiotherapy students served as the comparison group. The outcome variable of PI was calculated as a composite score with three subscales. In addition to sociodemographic data, potential determinants in cognitive, social, motivational, and behavioral dimensions were measured. Mixed effect models were used to analyze these determinants of PI formation.

**Results:**

The study included 276 participants. On average, PI in health promotion students was moderate and declined over the course of the undergraduate program. In contrast, PI in physiotherapy students was high from the beginning and remained stable throughout their studies. Factors such as gender, self-esteem, insecurity about the study program, the perceived social status of the profession, and planned behavior during and after the program were found to influence health promotion students’ PI formation.

**Discussion:**

Undergraduate health promotion students lack a strong PI, especially compared to physiotherapy students. Given the importance of a strong PI, the following interventions are suggested to strengthen health promotion students’ PI: (1) incorporating PI formation as a learning objective within curricula, and (2) enhancing the visibility and clarity of health promotion’s professional profile within undergraduate studies and in society. By recognizing the factors that shape PI and implementing targeted interventions, stakeholders can empower the next generation of health promotion practitioners to navigate their professional journeys with confidence and purpose, thereby strengthening workforce capacity building in health promotion.

## 1 Introduction

Professional identity (PI) is an important aspect of workforce capacity building. It is described as “the formation of an attitude of personal responsibility regarding one’s role in the profession, a commitment to behave ethically and morally, and the development of feelings of pride for the profession” (1). Therefore, PI leads to the successful adoption of the professional role, which is essential for quality awareness, ethical behavior, and a sense of professional commitment (2–4). In the field of public health, forming one’s PI is challenging due to the rapidly changing core themes, such as emergency preparedness for pandemics, wars, and the consequences of climate change (5–7). Furthermore, to develop a strong PI, the scope of the professional profile must be comprehensive and clear to professionals. This can be challenging due to the complexity of public health (8, 9). This is also true for health promotion practitioners, an emerging profession within the core public health workforce. Over the last 40 years, the ‘new public health’ movement has led to the establishment of educational programs specifically designed to train health promotion practitioners (11–13). While the traditional core public health workforce focuses on tasks such as food safety inspections and communicable disease prevention and monitoring, health promotion practitioners concentrate on planning, conducting, and evaluating health promotion programs in various settings, such as workplaces, schools, and cities (7). This study focuses on the newly evolving professional profile of health promotion practitioners.

### 1.1 The professional profile of health promotion practitioners

To define the professional profile of health promotion practitioners, scholars have described their roles, values, and professional developments over the past four decades (11–17) and have focused on capacity building for health promotion practitioners (18–24). A significant milestone in this effort is the development of the Core Competency Framework for Health Promotion (CompHP), which builds on the Ottawa Charter (25). The Ottawa Charter defines three major roles for health promotion, which remain relevant today in light of the sustainable development goals (26, 27): (1) *Advocate* for health at all policy levels and for all societal groups, recognizing that health is produced by socio-ecological factors; (2) *Enable* health equity for all by reducing disparities in health status and unequal opportunities and resources for health at the individual, societal and political levels; (3) *Mediate* in the pursuit of health among the differing interests of government, health, social, and economic sectors, as well as non-governmental and voluntary organizations, and local authorities. Although comprehensive, this description of the professional roles remains broad and complex, lacking a tangible scope.

The feasibility of defining a tangible scope for complex professional roles is demonstrated by health professions such as physiotherapy or medical doctors. Their professional roles are presumed to be clear and even legally regulated. Consequently, medical and physiotherapy students exhibit a strong sense of belonging to their professional group, which helps them form a strong PI from the beginning of their studies (28, 29). Studies have also shown that increased knowledge about the scope of their professions enhances PI formation (28). In contrast, the professional profile of health promotion practitioners is not yet well established or widely recognized by students or society (12, 14, 30). Additionally, there is limited research on the health promotion profession, particularly on the PI formation of the health promotion workforce (14, 31). This study aims to contribute to the understanding of PI formation among health promotion practitioners and to support the clarification of this emerging professional profile.

### 1.2 Professional identity formation among undergraduate students

PI is primarily formed during the transition from adolescence to adulthood, making it highly relevant to students (32–35). Educational institutions play a significant role in the PI formation of the future workforce. Recognizing this, scholars have begun to investigate PI formation in higher education across various professional sectors in order to identify ways to promote students’ PI formation (35–38). This is particularly beneficial, as a stronger PI is also associated with improved employment prospects (38). Additionally, PI facilitates the successful adoption of professional roles, which is crucial for quality assurance in practice (2–4). Beyond professional outcomes, a strong PI is linked to enhanced social support, better mental health, life satisfaction, and overall wellbeing (39–41).

To conceptualize PI formation, several approaches, adapted to the context of specific professions such as nursing and medicine, have been developed (34, 42, 43) and operationalized (28, 36, 37, 44, 45). Social psychological identity theories are particularly well suited to operationalizing PI formation as they aim to explain the dynamic development and the overall extent of PI. In this study we refer to the Social Identity Theory (36, 46, 47) and the identity-status model (48), which builds on Erikson’s work on identity development (49). They highlight that intra-individual as well as intergroup processes contribute to PI formation and that these influences change over time (36, 46, 47, 50, 51). At the intra-individual level, PI involves the motivational and cognitive efforts individuals make to align with their talents and abilities. This includes the central domains of identity and self-confidence derived from their professional choice (36, 50). At the intergroup level, the importance attributed to one’s professional group is emphasized. Individuals categorize themselves into social groups, leading to the formation of a collective identity (36, 46, 52). Both intra-individual and intergroup processes are substantial components of the PI outcome variable in this study, as described in the methods section.

### 1.3 Potential determinants of professional identity formation

Within the conceptualization of PI, a wide range of potential determinants of PI formation are discussed in the literature (29, 36, 38). Since there are no empirical findings on PI formation and its potential determinants in health promotion practitioners, we adopted a comprehensive and empirical approach, including a broad variety of determinants studied in students of other professions. We clustered these determinants into the following four dimensions to help us understand and predict the PI formation processes of health promotion practitioners.

*Cognitive*: PI formation is influenced by academic self-esteem and self-efficacy, which have been shown to predict career identity (32) and PI formation (36, 37). Conversely, insecurities about the professional choice or new professional fields can lead to stress and reduced commitment to professional goals, negatively impacting PI formation (35).

*Social*: Determinants of PI formation relate to an individual’s social environment. The degree to which individuals feel at ease with their peers or colleagues at work influences PI (53, 54). Additionally, approval of an individual’s professional choice from those around them (e.g., family and friends) positively correlates with PI (29, 55). Furthermore, a higher perceived social status of the profession is associated with a stronger PI (29, 56).

*Motivational*: Work values influencing PI formation have been investigated in various studies. Generally, intrinsic professional motivation (i.e., finding meaning through work) positively influences PI formation compared to extrinsic professional motivation (i.e., satisfying working conditions) (3, 36, 37, 57). Additionally, goal orientation or planned behavior regarding the study program or future career is assumed to positively influence PI formation (35). Conversely, a lack of motivation, measured by the intention to leave university, is associated with lower PI (36, 58).

*Behavioral*: Professional socialization is crucial within education and is a prerequisite for PI formation. This involves students learning about the attitudes, norms, and professional behavior of their professional group (e.g., specific networks, congresses, or journals) (29, 37, 38). Therefore, knowledge about the profession (59), work experiences or interactions with the profession (28, 29, 34, 36, 37), and existing role models (37) are essential for promoting PI formation.

### 1.4 Research gaps and research questions

As the existing literature on the professional profile of health promotion practitioners is predominantly descriptive and conceptual (11, 12, 14), this study develops a first understanding of a conceptual framework of PI formation and its determinants informed by social psychological identity theories and empirically explores the PI formation of health promotion relative to physiotherapy undergraduate students. Additionally, since longitudinal data on the potential determinants of PI formation in undergraduate health promotion students is lacking (31, 60), the present study assesses these determinants over time and analyzes their contribution. In summary, this study examines the following two research questions:

1) How do students’ professional identities develop during undergraduate health promotion and physiotherapy studies at a Swiss university?
2) Which determinants are relevant for the professional identity formation of undergraduate health promotion and physiotherapy students at a Swiss university?

## 2 Methods

### 2.1 Study design and participants

An observational longitudinal study was conducted in order to investigate the PI formation of undergraduate health promotion students over the course of their studies. The health promotion students represent the new professional profile of health promotion practitioners within the core public health workforce, which is the main focus of this study. Undergraduate physiotherapy students at the same Swiss university served as the comparison group.

Health promotion students were surveyed across four cohorts from 2016 to 2022. Only one cohort of undergraduate physiotherapy students, serving as the comparison group, was surveyed from 2016 to 2019. The online survey, conducted using the EVAsys survey tool, took place at three measurement points: at the beginning of the program (T1: first Semester), in the middle of the program (T2: fourth Semester), and at the end of the program (T3: sixth Semester). We have been able to assess PI formation of the first four cohorts of the entire population, as they were the first and only undergraduate health promotion students in Switzerland (61). The health promotion undergraduate program lasts 3 years and includes a six-month internship, typically completed at one institution during the sixth semester, between the second and third measurement points. The study sizes varied: 2016 (*n* = 45), 2017 (*n* = 36), 2018 (*n* = 52), and 2019 (*n* = 57). The control group was a convenience sample, selected for easy access and comparable conditions to the health promotion students in terms of location, lectures, and schedule (62). Additionally, this selection was also rationally validated by the existing comparative literature, which shows that PI in physiotherapy students is relatively strong from the beginning of their studies (28, 63). These factors help us interpret the findings related to the PI formation of health promotion students. The physiotherapy study program also lasts 3 years and includes three internships during the fourth and fifth semester, followed by a year of work placements before graduation. The 2016 physiotherapy study cohort consisted of 124 students.

All participants were informed in detail about the purpose of the research, data usage and the protection of their anonymity. They were also informed about their right to withdraw from the research at any time without providing a reason. Participants signed an informed consent form before participating in the study. Data was anonymized by using coded identifiers, which were securely stored on a separate server at the university. The study was conducted in accordance with the guidelines of the Declaration of Helsinki. The Ethics Committee of the Canton of Zurich approved a declaration of no objection (Req-2016-00711, 6th December 2016).

### 2.2 Measures

The following sociodemographic data and characteristics of the study sample were assessed: age, gender (male/female), place of origin (German/Italian/French speaking Switzerland/abroad), prior education (vocational/specialized baccalaureate, high school baccalaureate, bachelor’s/master’s and other), and study program as first choice. These variables, except the study program as first choice, were included as covariates in the mixed effect models, which are described in section 2.2.3, in order to control for potential confounders of PI formation.

#### 2.2.1 Outcome variable

The outcome variable was the extent of overall PI. As described in the introduction, we based our approach on social psychological theories of PI formation, operationalizing them in terms of intra-individual and intergroup domains. We used subscales from the Professional Identity Questionnaire by Mancini et al. (2015) and computed a mean score. PI was measured using the four-item subscales on the intra-individual level by “Identification with Commitment” (e.g., “Does thinking of yourself as a health promotion practitioner/ physiotherapist help you to understand who you are?”), and “Reconsideration of Commitment” (e.g., “Do you sometimes think that it would be better to study another profession?”), and on the intergroup level by “Affirmation” (e.g., “How important is it for you to become a health promotion practitioner/physiotherapist?”). Responses were assessed using a five-point Likert scale (“not at all” to “very much”). The reliability of the three subscales was high, with each Cronbach’s *α* ranging between 0.77 and 0.88. The subscales are reported in Table 1.

**Table 1:** Operationalization of the outcome variable and potential determinants of professional identity formation; German translation (GT).

Variables measured	Likert scale	No. of items	Reference of scales
Outcome variable			
Identification with commitment	1–5	4	(36) GT
Reconsideration of commitment	1–5	4	(29) GT
Affirmation	1–5	4	(29) GT
Potential determinants			
Cognitive			
Academic self-efficacy	1–6	7	(78) GT
Academic self-esteem	1–6	6	(79) GT
Insecurity regarding new study program (only applicable to health promotion students)	1–7	2	Authors developed items
Social			
Interpersonal attraction	1–6	3	(80) GT
Approval from family and friends	1–6	3	(55) GT
Perceived social status of the profession	1–7	3	(56) GT
Motivational			
Importance of different occupational characteristics (intrinsic and extrinsic professional motivation)	1–7	12	(81–83) GT
Intention to leave university	1–6	2	(29) GT
Planned behavior during and after studies	1–7	2	Items adapted from Ajzen and Fishbein (81)
Behavioral			
Knowledge about the profession	1–5	3	(59) GT
Experiences with the profession	1–5	3	(59) GT
Having a role model	1–5	3	(59) GT

#### 2.2.2 Potential determinants

As described in the introduction, we selected a wide range of potential determinants of PI formation, identified in the literature as relevant for students of other professions. All potential determinants measured as independent variables are shown in Table 1.

#### 2.2.3 Statistical analysis

For all variables, we computed frequency distributions, central tendencies, and standard deviations, and tested for skewness and normal distribution using histograms (62). Alpha level for statistical significance was set at *p* < 0.05.

Descriptive analyses were performed using the sociodemographic data, the characteristics of the study sample, the outcome variable, and the independent variables (means and standard deviations). Additionally, differences between professions for the outcome variable and potential determinants of PI formation were calculated using analyses of variance (ANOVAs).

Additionally, we needed an analysis method, which helps us to understand the PI formation in health promotion and physiotherapy students and its potential determinants over time. Mixed effect models were used to explain PI formation within individuals over time and across all study participants. These models extend common regression models by allowing certain regression coefficients to follow a random distribution, known as random effects. When analyzing longitudinal data such as those in the present study, participant-specific coefficients are specified as random effects, which makes it possible to account for intra-individual correlations over time (64–66). This accurately represents the hierarchical structure of longitudinal data and the associated error variance, enabling more precise data analysis (66). Separate models were calculated for health promotion and physiotherapy students in order to identify differences between professions and their determinants of PI formation. First, a model with covariates, including sociodemographic data, was calculated. Second, potential determinants were added. Lastly, a third model included interaction terms in order to identify potential moderating effects. Mixed effect models are suitable for the study’s exploratory and empirical approach, reducing alpha error inflation by including covariates, potential determinants, and selected interaction terms. Additionally, these models enable robust analysis of longitudinal data with missing values by considering both individual differences and systematic effects. The fixed effect component included dummies for time, gender, study cohort, prior education, study program as first choice, and the interaction between study cohort (only for health promotion students), gender and time. The last measurement point (time 3), male gender, the 2019 study cohort, and highest completed education were used as reference levels. The mixed effect models were estimated using the MIXED procedure, applying the REML method (restricted maximum likelihood) in SPSS version 29. For this reason, the data format was converted from wide to long format.

## 3 Results

### 3.1 Characteristics of the study population

**Figure 1:**
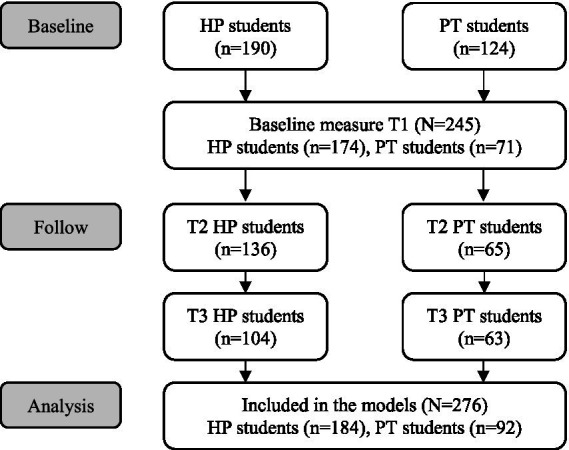
Consort flow diagram of the number of study participants (HP, health promotion; PT, physiotherapy).

Out of a population of 314 students, 276 participated in the study (88%). Health promotion students were from cohorts that started in 2016, 2017, 2018, and 2019, while physiotherapy students were from the 2016 cohort. Detailed participant descriptions are shown in Figure 1. The drop-out analysis revealed no substantial differences between the groups of participants with and without dropout. The response rate varied over the three measurement points and between professions. Health promotion students’ response rate decreased from over 90% at T1 to about 50% at T3. In contrast, physiotherapy students’ response rate remained relatively stable at a moderate level of about 55% across all three measurement points. Most participants were female (87.7%) and came from the German-speaking part of Switzerland (91.3%). Differences between professions were evident in prior vocational training, with 76.8% of health promotion students and 48.3% of physiotherapy students having completed such training. Further details can be found in Table 2.

**Table 2:** Sociodemographic information and characteristics of the study sample.

	Overall (*n* = 276)	Health promotion (*n* = 184, 66.7%)	Physiotherapy (*n* = 92, 33.3%)
Female gender, *n (%)*	242 (87.7)	165 (89.7)	77 (83.7)
Age, *mean, (SD)*	23.5 (4.5)	24.3 (4.8)	21.8 (3.3)
Times of measurement, *n* (%)			
T 1	245 (88.8)	174 (94.6)	71 (77.2)
T 2	201 (72.8)	136 (73.9)	65 (70.7)
T 3	167 (60.5)	104 (56.5)	63 (68.5)
Place of origin, *n* (%)			
German speaking Switzerland	251 (91.3)	166 (90.2)	85 (93.4)
Italian speaking Switzerland	4 (1.5)	2 (1.1)	2 (2.2)
French speaking Switzerland	2 (0.7)	0 (0.0)	2 (2.2)
Abroad	18 (6.5	16 (8.7)	2 (2.2)
Highest completed education, *n* (%)			
Vocational/ specialized baccalaureate	179 (67.3)	136 (76.8)	43 (48.3)
High school baccalaureate	72 (27.1)	31 (17.5)	41 (46.1)
Bachelor’s, master’s, other	15 (5.7)	5 (2.8)	5 (4.6)
Study program as first choice	120 (56.1)	60 (46.9)	60 (69.8)
Study cohort			
2016	–	45 (24.5)	92 (100)
2017	–	34 (18.5)	–
2018	–	52 (28.3)	–
2019	–	53 (28.8)	–

### 3.2 Research question 1: professional identity formation in undergraduate health promotion and physiotherapy students

**Figure 2:**
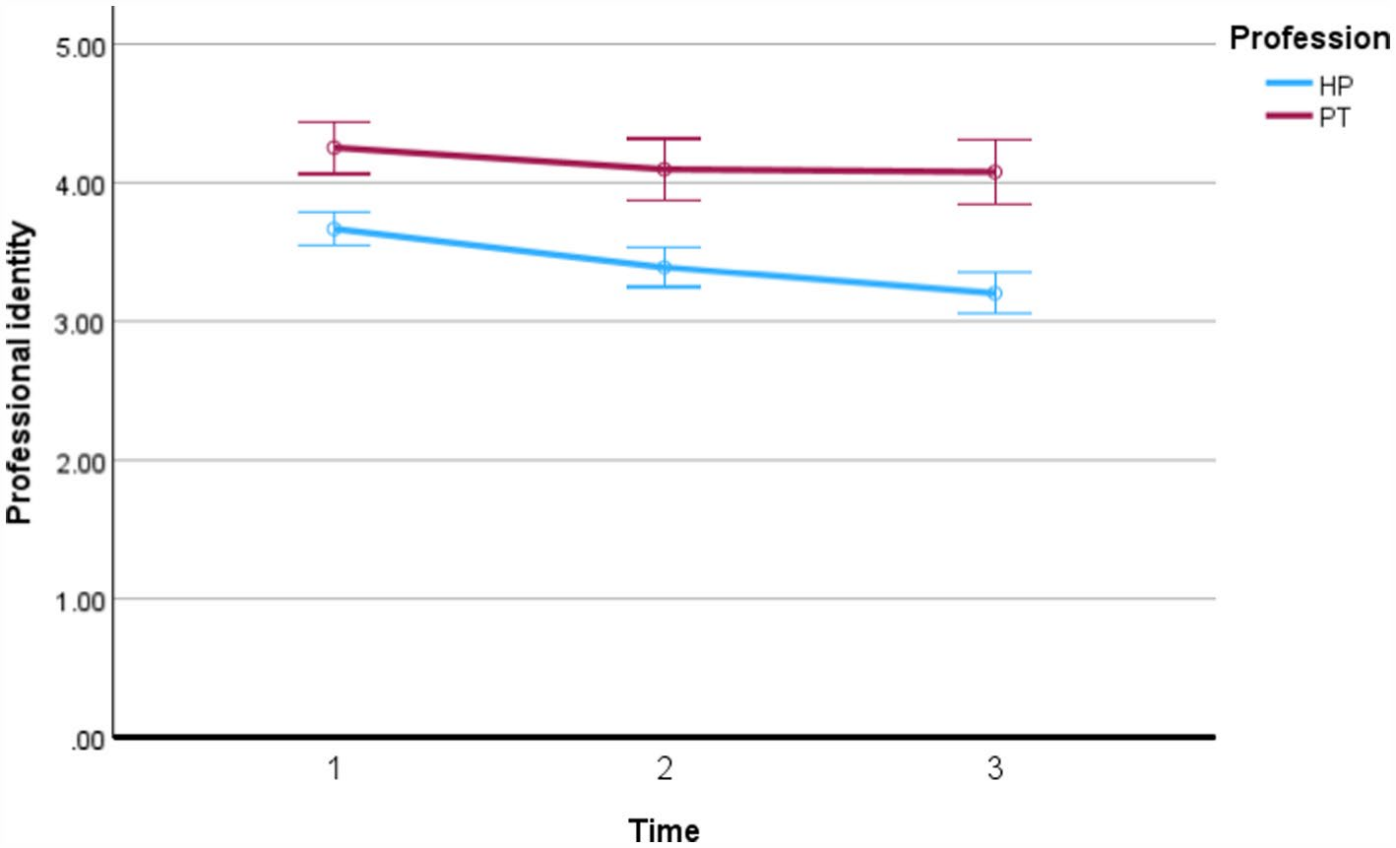
Professional identity formation by profession (HP, health promotion; PT, physiotherapy) over the course of study.

The descriptive analysis of the outcome variable PI indicates a significant decline from the first to the last semester among health promotion students. In contrast, physiotherapy students consistently rate their PI higher from the beginning and maintain stable ratings throughout their course (see Figure 2; Table 3). On both intra-individual and intergroup levels, the single items of PI formation show a steady decline in health promotion undergraduate students (Identification with commitment: T1: 3.27; T2: 3.08; T3: 2.78; Affirmation: T1: 4.01, T2: 3.78, T3: 3.60).

**Table 3:** Mean and standard deviation (SD) of the outcome and the independent variables reported for each time of measurement and profession.

Variables	Health promotion MW (SD)	Physiotherapy MW (SD)	ANOVA
Outcome variable			
Professional identity*			
T1 T2 T3	3.72 (0.62) 3.46 (0.76) 3.20 (0.77)	4.11 (0.64) 4.05 (0.47) 4.04 (0.58)	*F*(1, 242) = 19.303, *p* < 0.001 *F*(1, 198) = 31.980, *p* < 0.001 *F*(1, 160) = 26.967, *p* < 0.001
Independent variables			
Cognitive			
Self-esteem (1–6)			
T1 T2 T3	4.89 (0.68) 4.92 (0.75) 4.85 (0.82)	4.84 (0.80) 4.95 (0.72) 5.01 (0.78)	*F*(1,243) = 0.242, *p* = 0.623 *F*(1,197) = 0.079, *p* = 0.779 *F*(1, 158) = 1.530, *p* = 0.218
Self-efficacy (1–6)*			
T1 T2 T3	4.36 (0.85) 4.87 (0.65) 5.12 (0.57)	4.12 (0.85) 4.36 (0.66) 4.55 (0.69)	*F*(1, 243) = 4.312, *p* = 0.039 *F*(1, 196) = 17.617, *p* < 0.001 *F*(1, 158) = 32.274, *p* < 0.001
Insecurity regarding new study program (1–7)			
T1 T2 T3	3.11 (1.23) 3.53 (1.24) 3.79 (1.48)	– – –	– – –
Social			
Interpersonal attraction (1–6)*			
T1 T2 T3	4.21 (1.04) 4.54 (0.93) 4.44 (1.13)	4.85 (0.78) 4.80 (0.90) 4.78 (0.96)	*F*(1,242) = 21.122, *p* < 0.001 *F*(1, 198) = 3.343, *p* = 0.069 *F*(1, 160) =3.838, *p* = 0.052
Approval from family and friends (1–6)*			
T1 T2 T3	4.86 (0.86) 4.67 (0.83) 4.07 (1.41)	5.67 (0.53) 5.66 (0.45) 5.55 (0.64)	*F*(1, 243) = 53.957, *p* < 0.001 *F*(1, 198) = 79.342, *p* < 0.001 *F*(1, 161) = 59.358, *p* < 0.001
Perceived social status of the profession (1–7)*			
T1 T2 T3	5.40 (0.82) 5.07 (0.84) 4.80 (1.01)	5.93 (0.62) 5.75 (0.73) 5.96 (0.72)	*F*(1, 243) = 23.761, *p* < 0.001 *F*(1, 197) = 31.056, *p* < 0.001 *F*(1, 160) = 60.762, *p* < 0.001
Motivation			
Intrinsic professional motivation (1–7)*			
T1 T2 T3	6.31 (0.43) 6.18 (0.50) 6.19 (0.50)	6.39 (0.42) 6.27 (0.40) 6.29 (0.37)	*F*(1, 243) = 1.698, *p* = 0.194 *F*(1, 198) = 1.714, *p* = 0.192 *F*(1, 160) = 1.713, *p* = 0.192
Extrinsic professional motivation (1–7)			
T1 T2 T3	5.93 (0.72) 5.80 (0.75) 5.74 (0.67)	5.33 (0.78) 5.50 (0.80) 5.65 (0.67)	*F*(1, 243) = 33.334, *p* < 0.001 *F*(1, 198) = 6.564, *p* = 0.011 *F*(1, 160) = 0.658, *p* = 0.409
Intention to leave university (1–6)*			
T1 T2 T3	2.05 (1.30) 2.18 (1.42) 2.61 (1.57)	1.51 (1.08) 1.45 (0.74) 1.55 (0.15)	*F*(1, 242) = 9.540, *p* = 0.002 *F*(1, 198) = 14.778, *p* < 0.001 *F*(1, 161) = 20.773, *p* < 0.001
Planned behavior during and after studies (1–7)*			
T1 T2 T3	3.81 (1.47) 3.71 (1.61) 4.06 (2.16)	4.28 (1.42) 4.53 (1.49) 4.75 (1.67)	*F*(1, 243) = 5.283, *p* = 0.022 *F*(1, 196) = 11.701, *p* < 0.001 *F*(1, 160) = 4.534, *p* = 0.035
Behavior			
Knowledge about the profession (1–5)*			
T1 T2 T3	2.92 (0.75) 3.43 (0.76) 3.75 (0.74)	3.99 (0.61) 4.14 (0.45) 4.21 (0.59)	*F*(1, 243) = 114.074, *p* < 0.001 *F*(1, 198) = 21.890, *p* < 0.001 *F*(1, 160) = 17.469, *p* < 0.001
Experiences with the profession (1–5)*			
T1 T2 T3	2.61 (0.93) 3.12 (0.94) 3.54 (1.01)	3.80 (0.86) 3.83 (0.71) 3.91 (0.81)	*F*(1, 242) = 85.127, *p* < 0.001 *F*(1, 197) = 29.067, *p* < 0.001 *F*(1, 160) = 5.859, *p* = 0.017
Having a role model (1–5)*			
T1 T2 T3	3.14 (0.71) 3.31 (0.79) 3.38 (0.67)	3.61 (0.59) 3.53 (0.71) 3.61 (0.69)	*F*(1, 241) = 24.267, *p* < 0.001 *F*(1, 181) = 3.503, *p* = 0.063 *F*(1, 159) = 4.331, *p* = 0.039

Differences between professions calculated with ANOVA; *significant differences between times of measurement calculated with repeated measures analysis of variance (*p* < 0.05).

### 3.3 Research question 2: potential determinants of professional identity formation in undergraduate health promotion and physiotherapy students

Before analyzing the effect of potential determinants on PI formation, we calculated the differences between the health promotion and physiotherapy students in these determinants, as detailed in Table 3. On the cognitive dimension, health promotion students report higher self-efficacy compared to physiotherapy students. A clear difference is also observed on the social dimension, with health promotion students receiving lower approval from family and friends and perceiving the profession to be of lower social status than physiotherapy students. Furthermore, on the motivational dimension, health promotion students were more likely to declare their intention to leave university at all three measurement points, while physiotherapy students had clearer plans during and after their studies. On the behavioral dimension, physiotherapy students rate higher than health promotion students in knowledge about the profession, experience with the profession, and having a role model.

To identify the potential determinants of PI formation, we calculated mixed effect models for health promotion and physiotherapy students separately. Overall, PI formation of health promotion students reveals more potential determinants and the models show better fit (Model 3: *R*^2^ = 0.679) compared to physiotherapy students (Model 3: *R*^2^ = 0.506). Detailed results are provided in Table 4, with some highlights mentioned here. For health promotion students, when only the covariates are included, significant main effects were observed for study cohort (*F* = 3.099, *p* = 0.028), gender (*F* = 4.053, *p* = 0.046), and time (*F* = 18.415, *p* < 0.001). No significant effects of the covariates on PI formation were identified for physiotherapy students.

**Table 4:** Results of the three mixed effect models with professional identity as the outcome variable.

Variables	Health promotion			Physiotherapy		
	Model 1	Model 2	Model 3	Model 1	Model 2	Model 3
R^2^ marginal	0.131	0.668	0.679	0.026	0.507	0.506
ICC adapted	0.287	0.176	0.194	0.385	0.229	0.222
AIC	840.922	417.301	412.789	326.554	243.302	245.760
Covariates						
Study cohort	*F* = 3.099, *p* = 0.028	*F* = 0.587, *p* = 0.625	*F* = 0.242, *p* = 0.867	–	–	–
2016	*B* = −0.285 (0.116)*	*B* = 0.061 (0.144)	*B* = 0.155 (0.376)	–	–	–
2017	*B* = −0.174 (0.128)	*B* = 0.156 (0.153)	*B* = 0.224 (0.444)	–	–	–
2018	*B* = 0.010 (0.113)	*B* = 0.097 (0.146)	*B* = 0.067 (0.319)	–	–	–
2019 (Ref)	–	–	–	–	–	–
Gender	*F* = 4.053, *p* = 0.046	*F* = 9.339, *p* = 0.003	*F* = 8.489, *p* = 0.004	*F* = 0.174, *p* = 0.678	*F* = 0.295, *p* = 0.589	*F* = 0.360, *p* = 0.550
Female	*B* = −0.281 (0.139)*	*B* = −0.299 (0.098)*	*B* = 0.0–0.383 (0.200)	*B* = 0.060 (0.143)	*B* = −0.056 (0.102)	*B* = −0.144 (0.163)
Male (Ref)	–	–	–	–		
Time of measurement	*F* = 18.415, *p* < 0.001	*F* = 4.628, *p* = 0.011	*F* = 0.867, *p* = 0.422	*F* = 0.815, *p* = 0.446	*F* = 0.515, *p* = 0.599	*F* = 0.351, *p* = 0.705
T 1	*B* = 0.456 (0.076)*	*B* = 0.213 (0.082)*	*B* = 0.069 (0.386)	*B* = 0.092 (0.082)	*B* = 0.074 (0.075)	*B* = 0.002 (0.186)
T 2	*B* = 0.217 (0.079)*	*B* = 0.032 (0.069)	*B* = 0.582 (0.408)	*B* = 0.004 (0.084)	*B* = 0.020 (0.069)	*B* = −0.124 (0.188)
T 3 (Ref)	–	–	–	–	–	–
highest completed education	*F* = 1.029, *p* = 0.360	*F* = 0.531, *p* = 0.589	*F* = 0.461, *p* = 0.632	*F* = 1.078, *p* = 0.346	*F* = 0.381, *p* = 0.685	F = 0.381, *p* = 0.685
vocational/ specialized baccalaureate	*B* = 0.016 (0.179)	*B* = −0.159 (0.157)	*B* = −0.150 (0.157)	*B* = −0.242 (0.263)	*B* = −0.149 (0.181)	*B* = −0.153 (0.181)
high school baccalaureate	*B* = −0.139 (0.199)	*B* = −0.032 (0.069)	*B* = −0.150 (0.165)	*B* = −0.107 (0.266)	*B* = −0.117 (0.183)	*B* = −0.127 (0.183)
Bachelor, master, other (Ref)	–	–	–	–	–	–
Age	*F* = 1.213, *p* = 0.272 *B* = 0.011 (0.010)	*F* = 0.002, *p* = 0.961 *B* = 0.000 (0.008)	*F* = 0.006, *p* = 0.938 *B* = 0.001 (0.008)	*F* = 0.039, *p* = 0.843 *B* = –0.004 (0.020)	*F* = 0.047, *p* = 0.828 *B* = −0.003 (0.014)	*F* = 0.073, *p* = 0.788 *B* = −0.004 (0.014)
Potential determinants						
Study program as first choice	–	*F* = 1.822, *p* = 0.180	*F* = 2.145, *p* = 0.146		*F* = 0.273, *p* = 0.603	*F* = 0.215, *p* = 0.644
Yes	–	*B* = 0.080 (0.059)	*B* = 0.088 (0.060)		*B* = −0.046 (0.087)	*B* = −0.041 (0.088)
No (Ref)	–	–	–		–	–
Cognitive						
Self-esteem		*F* = 15.026, *p* < 0.001 *B* = 0.183 (0.047)*	*F* = 15.21, *p* < 0.001 *B* = 0.207 (0.053)*		*F* = 5.357, *p* = 0.022 *B* = 0.147 (0.063)*	*F* = 5.535, *p* = 0.020 *B* = 0.151 (0.064)*
Self-efficacy		*F* = 4.583, *p* = 0.033 *B* = −0.089 (0.041)*	*F* = 4.509, *p* = 0.035 *B* = −0.089 (0.042)*		*F* = 0.392, *p* = 0.532 *B* = −0.038 (0.061)	*F* = 0.378, *p* = 0.540 *B* = −0.038 (0.061)
Insecurity regarding the new study program (only health promotion)		*F* = 23.809, *p* < 0.001 *B* = −0.106 (0.022)*	*F* = 18.341, *p* < 0.001 *B* = −0.098 (0.023)*		–	–
Social						
Interpersonal attraction		*F* = 1.827, *p* = 0.178 *B* = 0.041 (0.031)	*F* = 1.010, *p* = 0.316 *B* = 0.031 (0.031)		*F* = 0.338, *p* = 0.562 *B* = 0.024 (0.041)	*F* = 0.330, *p* = 0.567 *B* = 0.024 (0.041)
Approval from family and friends		*F* = 0.614, *p* = 0.434 *B* = −0.024 (0.030)	*F* = 0.061, *p* = 0.805 *B* = 0.0–0.009 (0.034)		*F* = 0.293, *p* = 0.589 *B* = 0.033 (0.061)	*F* = 0.193, *p* = 0.661 *B* = 0.027 (0.062)
Perceived social status of the profession		*F* = 12.949, *p* < 0.001 *B* = 0.129 (0.036)*	*F* = 10.205, *p* = 0.002 *B* = 0.117 (0.036)*		*F* = 1.1723, *p* = 0.192 *B* = 0.063 (0.048)	*F* = 1.807, *p* = 0.181 *B* = 0.065 (0.048)
Motivational						
Intrinsic professional motivation		*F* = 0.542, *p* = 0.462 *B* = −0.050 (0.068)	*F* = 1.313, *p* = 0.253 *B* = −0.078 (0.068)		*F* = 0.148, *p* = 0.701 *B* = 0.034 (0.089)	*F* = 0.240, *p* = 0.625 *B* = 0.044 (0.090)
Extrinsic professional motivation		*F* = 2.802, *p* = 0.095 *B* = 0.070 (0.042)	*F* = 3.789, *p* = 0.053 *B* = 0.082 (0.042)		*F* = 0.024, *p* = 0.878 *B* = −0.007 (0.044)	*F* = 0.048, *p* = 0.827 *B* = −0.010 (0.044)
Intention to leave university		*F* = 91.245, *p* < 0.001 *B* = −0.227 (0.024)*	*F* = 82.653, *p* < 0.001 *B* = −0.226 (0.025)*		*F* = 69.403, *p* < 0.001 *B* = −0.329 (0.049)*	*F* = 66.513, *p* < 0.001 *B* = −0.328 (0.040)*
Planned behavior during and after studies		*F* = 3.332, *p* = 0.069 *B* = 0.030 (0.017)	*F* = 2.194, *p* = 0.140 *B* = 0.025 (0.017)		*F* = 0.065, *p* = 0.799 *B* = −0.006 (0.025)	*F* = 0.054, *p* = 0.817 *B* = −0.006 (0.025)
Behavioral						
Knowledge about the profession		*F* = 0.084, *p* = 0.773 *B* = 0.013 (0.044)	*F* = 0.007, *p* = 0.932 *B* = 0.004 (0.044)		F = 0.002, *p* = 0.962 *B* = 0.003 (0.073)	*F* = 0.011, *p* = 0.916 *B* = −0.008 (0.075)
Experiences with the profession		*F* = 0.133, *p* = 0.716 *B* = −0.012 (0.032)	*F* = 0.239, *p* = 0.625 *B* = −0.016 (0.032)		*F* = 1.689, *p* = 0.196 *B* = 0.062 (0.048)	*F* = 1.803, *p* = 0.181 *B* = 0.065 (0.048)
Having a role model		*F* = 3.210, *p* = 0.074 *B* = 0.074 (0.041)	*F* = 2.580, *p* = 0.109 *B* = 0.067 (0.042)		*F* = 0.168, *p* = 0.683 *B* = 0.025 (0.060)	*F* = 0.160, *p* = 0.689 *B* = 0.025 (0.061)
Study cohort*gender			*F* = 0.087, *p* = 0.916			–
Gender* time			*F* = 0.936, *p* = 0.394			*F* = 0.344, *p* = 0.710
Study cohort* time			F = 3.031, *p* = 0.007			–
2016*time1			*B* = −0.017 (0.351)			–
2016*time2			*B* = −0.639 (0.374)			–
2016*time3 (Ref)			–			–
2017*time1			*B* = −0.018 (0.385)			–
2017*time2			*B* = −0.640 (0.395)			–
2017*time3 (Ref)			–			–
2018*time1			*B* = 0.093 (0.350)			–
2018*time2			*B* = −0.157 (0.373)			–
2018*time3 (Ref)			–			–
2019 (Ref)			–			–

Model 1 includes the covariates; Model 2 adds all potential determinants of professional identity formation; Model 3 adds selected interaction terms. The models were calculated separately for health promotion (*n* = 174) and physiotherapy students (*n* = 71). *p< 0.05.

To estimate the potential determinants of PI formation, we report the results of model 3, which includes covariates, the outcome variable, and selected interaction terms. For health promotion students, the interaction between study cohort and time is significant (*F* = 3.031, *p* = 0.007), indicating that each cohort has its own specific process of PI formation. Students from the 2016 and 2017 cohorts score lower on PI at every measurement point compared to the 2018 and 2019 cohorts. Regarding the potential determinants of PI formation, effects on the cognitive, social, and motivational dimension become relevant. On the cognitive dimension, self-esteem (*F* = 15.21, *p* < 0.001), self-efficacy (*F* = 4.509, *p* = 0.035) and insecurity regarding the study program (*F* = 18.341, *p* < 0.001) are significant determinants. For physiotherapy students, higher self-esteem also positively determines PI formation (*F* = 5.535, *p* = 0.020).

Regarding the social dimension, the data reveals that a low perceived social status of the profession weakens the PI of health promotion students (*F* = 10.205, *p* = 0.002). For physiotherapy students, the social dimension does not appear to be relevant for PI formation.

On the motivational dimension, the intention to leave university negatively influences PI formation for both professions (health promotion: *F* = 82.653, *p* < 0.001; physiotherapy: *F* = 66.513, *p* < 0.001). No further potential determinants, including those on the behavioral dimension, significantly influence PI formation for either health promotion or physiotherapy students.

## 4 Discussion

PI is crucial for quality assurance in professional practice, as it fosters the adoption of professional roles, commitment to the profession, high engagement, and motivation (2–4). Within designated educational programs, health promotion practitioners acquire specific competencies, leading to the development of a new professional profile within the core public health workforce (7, 11, 12). A primary objective of these programs is to enable students to form their PI, given its importance for quality assurance in professional practice (60, 67, 68). To promote PI formation among health promotion practitioners, it is essential to understand the complex process that begins in educational programs. The study provides a comprehensive exploration of the multifaceted processes of PI formation and its potential determinants among undergraduate health promotion students, compared to physiotherapy students.

### 4.1 Professional identity formation among undergraduate health promotion and physiotherapy students

Regarding research question one, which examines how students’ PIs develop during undergraduate health promotion and physiotherapy studies at a Swiss university, our data analysis reveals a concerning trend: PI is moderate at the beginning of the program and declines steadily among health promotion students throughout their academic journey. However, the first two study cohorts (2016 and 2017) have lower PI scores than the 2018 and 2019 cohorts. This effect might suggest that the number of times the study program is delivered influences the PI level of health promotion students, as both students and lecturers become more confident with the study program. Feedback from practice or graduates reaffirms the relevance of the health promotion practitioner and the alignment of the undergraduate studies with practice. In general, our results confirm the assumption of social psychological identity theories that PI formation is not stable but a dynamic construct. The literature supports the view that PI formation is a highly individual, complex, and context-specific journey, which is not linear and requires a deeper understanding beyond professional values, individual characteristics, or behaviors (45, 69). In our sample of health promotion students, both the intra-individual level (relating to the central domains of identity and self-confidence derived from the choice of profession) and the intergroup level (relating to the importance attributed to one’s professional group) decline over the course of study.

In addition to these findings on the decline of PI among health promotion students over the course of their studies, the analysis also provides insights into the differences between health promotion and physiotherapy students. This comparison reveals a significant discrepancy in PI levels, with health promotion students recording lower PI scores. This difference might be attributed to a clearer understanding of professional roles and a deeper knowledge base within the physiotherapy field (28), as shown in this study. Although PI appears to be stronger among physiotherapy students in our sample, it remains uncertain how their PI will develop in the job market. The healthcare workforce, including physiotherapy, faces multiple crises, one of which is career retention (70, 71). Further research should investigate how the PI of physiotherapies develops in the job market and whether promoting physiotherapy’s PI could prevent career termination.

### 4.2 Potential determinants of professional identity formation in undergraduate health promotion and physiotherapy students

Concerning research question two, which examines the potential determinants of importance for the PI formation of undergraduate health promotion and physiotherapy students at a Swiss university, the study uncovers cognitive, motivational, and social determinants of PI formation. These determinants differ between health promotion and physiotherapy students, with the determinants better predicting health promotion students’ PI than that of physiotherapy students. One potential explanation is a ceiling effect in the data for physiotherapy students, as they consistently rate their PI as very high over the three measurement points. This lack of variance may limit the explainability of the independent variables in the mixed effects models for physiotherapy students. Moreover, since PI formation is influenced by a variety of personal and contextual factors that may differ between professions (29, 45, 69), our limited selection of potential determinants may better fit the situation of health promotion students than that of physiotherapy students. Further research is needed to improve our understanding of how these differences between the professions in PI formation and its potential determinants arise.

As an unexpected determinant, gender emerged as a significant factor in PI formation among health promotion students, with men displaying stronger PI than women. This contrasts with previous research, which found that women typically have stronger PI than their male counterparts (28). These findings might arise due to the small male sample in the present study, as only about 10% of health promotion students are male. These students might represent a particularly motivated, self-selected group. Although the inconsistent findings cannot yet be resolved, the gender disparity underscores the need to address systemic biases and promote gender equality within the health promotion profession, starting at the education level. This issue has already been addressed in various studies of other predominantly female-driven health professions, such as nursing, occupational therapy, and social work (70, 72). Addressing gender disparities and promoting inclusivity within the health promotion profession are essential steps toward creating a supportive and equitable environment for all aspiring health promotion practitioners and retaining a diverse talent pool that reflects the complexity and richness of the communities health promotion serves.

Further determinants of PI formation among health promotion students were identified on the cognitive, social, and motivational dimensions, aligning with prior findings. These include higher levels of self-esteem (36, 37), the perceived societal status of the profession (29, 56, 73), and planned behavior during and after studies (35). These findings highlight the importance of cognitive, social and motivational dimensions in shaping the PI of health promotion practitioners. An interesting insight is that the perceived social status of the profession is a significant determinant of PI formation in health promotion students. Students perceive their profession to be poorly acknowledged because the professional profile is new and not well known in Switzerland. This is in line with social psychological identity theories, which emphasize that a sense of belonging to a professional group that is positively evaluated is crucial for PI formation (36, 47). However, health promotion students lack this societal recognition, which is detrimental to their PI development.

Conversely, intentions to leave the university, higher self-efficacy, and insecurity regarding professional choice are linked to lower PI, emphasizing the influence of personal beliefs and career intentions on PI formation. Contrary to the existing literature, our study found that higher self-efficacy is associated with lower PI in health promotion students (36, 37). Another study investigating the PI of social work students suggests that students with high self-efficacy need to experience openness in their training programs and professional practice in order to support their PI formation (74). Based on this finding, we could conclude that the health promotion students with high self-efficacy in our sample experienced a lack of flexibility and openness in the educational program and their professional practice, e.g., in their internships. Focus group results with the same health promotion students show that some students experience a mismatch between practice and the theory taught in the educational program (60). Additionally, some students report a non-welcoming atmosphere in the professional community, which poses a significant barrier to PI formation (60). These aspects might explain the reversed effect of high self-efficacy in low PI.

Generally, the findings confirm the relevance of the many cognitive, motivational and social determinants previously described in the literature (see 1.3). However, determinants within the behavioral dimension did not significantly impact PI formation in our sample. Instead, the study highlights the importance of experiences with the educational program and in professional practice (e.g., internships), which were not included in the questionnaire. Consequently, these findings suggest that the initial conceptual framework informed by social psychological identity theories should be further refined in order to better operationalize PI determinants by incorporating these educational and practical experiences.

### 4.3 Practical implications of the study’s results

The study’s results contribute to our understanding of PI formation among health promotion practitioners and may potentially be used to support the professional development of this newly established professional profile. Two practical implications are derived for the health promotion workforce in order to address the decline of PI over the course of study and the difficulty in grasping the professional profile of health promotion practitioners: (1) incorporate PI formation as a learning objective within curricula; (2) make the health promotion professional profile clearer and more visible. Both suggested interventions align with social psychological theories on PI formation that emphasize the importance of strengthening the sense of belonging to a professional group and of establishing superordinate goals as a professional group as a means to strengthen group identification.

Given that our data revealed a decline in PI among undergraduate health promotion students over the course of study, the first intervention involves educators and practitioners leveraging PI formation by designing educational curricula and professional development programs that foster a deeper understanding of professional roles, enhance self-esteem, and promote a sense of belonging to the profession (6, 10, 29, 31, 75). Blackford and colleagues implemented work-integrated learning in the curriculum of undergraduate health promotion students in order better to reflect their professional roles throughout the curriculum. It is important to implement specific workshops or modules from the beginning of the course, adopt PI formation as an educational objective, and make this visible to students (6, 29, 35).

The second practical implication involves enhancing the recognition, visibility, and credibility of the profession by clarifying and credentialing the professional profile of health promotion practitioners and making the scope of the professional profile tangible. It is important to develop clear narratives for the health promotion professional profile and to clarify its distinctive profile, even within the public health workforce. Establishing professional certification and accreditation mechanisms can ensure quality standards and competencies. Certified professionals are more likely to be recognized and trusted by employers, policymakers, and the public, as suggested in the WHO’s “Roadmap to professionalizing the public health workforce in the European region” (7). Credentialing the profession can help raise the visibility of the professional profile (12, 31, 76, 77). Effective approaches to achieve this might include advocacy campaigns and raising public awareness about the importance of health promotion as well as the role of health promotion practitioners in improving individual and community wellbeing (19, 20). This effort could potentially be undertaken by professional associations such as the IUHPE, which is already engaged in such activities. However, such associations are rather scarce at the national level. Conducting research to understand the professionalization of the health promotion and public health workforce and engaging with professional associations can elevate the professional profile of health promotion practitioners and position them as leaders in the field. By collectively implementing these strategies, stakeholders can enhance recognition of the profession’s importance, and ultimately contribute to improved health outcomes for individuals and communities by strengthening the PI of health promotion practitioners.

### 4.4 Limitations

This study has several limitations that impact the generalizability and robustness of the findings. The research design included multiple cohorts for the health promotion component but only a single cohort for the physiotherapy component. This imbalance limits the comparative analysis between these fields and reduces the ability to draw broad conclusions about the differences or similarities in the development of PI across diverse health professions. We chose this approach in order to obtain a larger sample of health promotion students by including four cohorts. However, the sample size was still insufficient to achieve strong statistical power, constraining the ability to generalize findings to the wider population of health promotion and physiotherapy professionals. A larger sample would provide a more representative understanding of the processes involved in PI formation and the effectiveness of health promotion education programs. In Switzerland, there is only one undergraduate program in health promotion. Therefore, an international comparison would add value by providing insight into the PI formation of undergraduate health promotion students.

Moreover, measuring PI formation was challenging due to the lack of a clear conceptual boundary distinguishing the concept of PI from its potential determinants, such as personal values, educational experiences, and workplace culture. This overlap makes it difficult to isolate the specific elements that contribute to PI formation and assess them independently. The highly individual process of PI formation suggests that the operationalization used in this study may not fully capture the PI of undergraduate health promotion and physiotherapy students. Recently, further assessment tools have been developed that include qualitative approaches or reflective writing as methods to measure PI formation (45). These methods allow for a deeper understanding of PI formation and its potential determinants. Our study suggests that the assessment tool should be adapted to include qualitative methods in future investigations of PI formation.

## 5 Conclusion

This longitudinal study offers valuable insights into the intricate process of PI formation among undergraduate health promotion and physiotherapy students and potentially provides support for the professional development of the newly established health promotion professional profile. The results show a decline in PI among health promotion students over their course of study, indicating challenges in grasping the professional profile and incorporating it into their self-concept. Therefore, PI formation in health promotion students needs to be promoted throughout their studies, and potential challenges, such as the low perceived social status of the health promotion professional profile, need to be addressed. By recognizing the factors that shape PI formation and implementing targeted interventions, stakeholders can empower the next generation of health promotion practitioners to navigate their professional journeys with confidence and purpose. The interventions discussed are twofold: (1) incorporating PI formation as a learning objective within curricula, and (2) making the health promotion professional profile clearer and more visible. The process of PI formation is highly individual, influenced by various personal and contextual factors, and is not completed within undergraduate education. The study’s empirical approach makes it possible to refine the initial conceptual framework informed by social psychological identity theories and its operationalizations for PI formation and its potential determinants among undergraduate health promotion students. Regarding the operationalization, we advocate considering both intra-individual and intergroup processes of PI formation. The study’s results highlight the importance of a sense of belonging to a professional group, which remains fragile in health promotion. More research is needed to understand the role of the health promotion professional community in PI formation for health promotion students and graduates. Additionally, we recommend adjusting the method used to assess PI formation by including qualitative methods. Regarding the potential determinants, we find that the selected independent variables are well suited to the health promotion student sample but suggest omitting or adapting the behavioral dimension and integrating students’ experience with the educational program and their professional practice as potential determinants of PI formation. Further research is needed on the PI formation of undergraduate health promotion students, building on this study’s findings and implications regarding the conceptual framework and its operationalization. Moreover, in the meantime several cohorts of health promotion undergraduate students have completed their studies, and the health promotion study program has been updated, making it less novel than during the study. Therefore, it would be interesting to examine the PI formation process in the current health promotion study cohorts. We would expect that PI has improved as more of the recommendations concerning the fostering of the PI formation have been implemented. Additionally, examining how PI evolves post-education is critical for designing interventions that support ongoing professional development. Furthermore, understanding how health promotion practitioners without explicit formal education develop their PI, the values and professional norms they rely on, and how these might differ from those with formal education can provide deeper insights.

In sum, this study shows that ensuring the development of a competent and motivated workforce, and therefore capacity building in health promotion and public health as a whole, is a crucial topic in health promotion education. However, our understanding of PI formation among health promotion practitioners needs to be further improved, as it contributes to more effective professional practice and, consequently, to better health outcomes.

## Data Availability

The raw data supporting the conclusions of this article will be made available by the authors, without undue reservation.
